# Positive Association between *EDN1* rs5370 (Lys198Asn) Polymorphism and Large Artery Stroke in a Ukrainian Population

**DOI:** 10.1155/2018/1695782

**Published:** 2018-04-03

**Authors:** Yevhen I. Dubovyk, Tetyana B. Oleshko, Viktoriia Yu. Harbuzova, Alexander V. Ataman

**Affiliations:** ^1^Department of Physiology, Pathophysiology and Medical Biology, Sumy State University, Sumy 40007, Ukraine; ^2^Scientific Laboratory of Molecular Genetic Research, Sumy State University, Sumy 40007, Ukraine

## Abstract

There are a lot of convincing evidences about the involvement of endothelin pathway proteins in the pathogenesis of atherosclerosis and its fatal complications. In this study, the analysis of a possible association between *EDN1* rs5370 and *EDNRA* rs5335 gene polymorphisms and the risk of large artery stroke (LAS) in a Ukrainian population was conducted. 200 LAS patients and 200 unrelated controls were enrolled in a case-control study. The polymerase chain reaction-restriction fragment length polymorphism method (PCR-RFLP) was used for SNP genotyping. Our results revealed that *EDN1* rs5370 polymorphism was associated with LAS development both before and after adjustment for atherosclerosis risk factors (sex, age, body mass index, arterial hypertension, type 2 diabetes mellitus, and smoking). The risk for a LAS incident in rs5370-T allele carriers was 1.6 times higher (CI = 1.066–2.403; *P* = 0.020) than in subjects with the GG genotype. No link between *EDNRA* rs5335 and LAS risk in a Ukrainian population was found. The present study indicated that *EDN1* rs5370, but not *EDNRA* rs5335, can be the strong genetic predictor for LAS development in a Ukrainian population.

## 1. Introduction

As it is well known, endothelial dysfunction plays an important role in the development of common cardiovascular diseases and their complications [1]. One of the main pathogenetic pathways of endothelial dysfunction development is an increased formation and biological activity of the powerful vasoconstrictor and proinflammatory peptide endothelin (ET-1) [2], which mediates own effects via two pharmacologically distinguishable receptor subtypes, endothelin A (ETA) and endothelin B (ETB) receptors, respectively [3]. There are several lines of evidence indicating that the ET-1-induced endothelial dysfunction is realized through decreasing production and increasing degradation of NO, through enhancement of Von Willebrand factor and reactive oxygen species formation, and also through the activation of proinflammatory metabolic pathways in the endotheliocytes [4].

In recent years, a wide range of case-control studies to test the association between various single nucleotide polymorphisms (SNPs) of the ET-1 (*EDN1*) and its receptor (*EDNRA* and *EDNRB*) genes and development of arterial hypertension (AH) [5, 6], pulmonary hypertension [7], myocardial infarction [8], diabetic retinopathy [9] and nephropathy [10], metabolic syndrome [11], and hemorrhagic stroke [12] have been carried out. There are several works that concern the investigation of the effect of *EDN1*, *EDNRA*, and *EDNRB* genetic polymorphisms on ischemic stroke (IS) development. Zhang and Sui showed that rs5370-T allele (*EDN1* gene) increased the IS incidence risk in Northern Han men, whereas the rs5335-CC genotype (*EDNRA* gene) had a protective effect in the same population [13]. Yamaguchi et al. revealed a significant association between *EDN1* rs5370 polymorphism and high risk for IS only among Japanese women [14], whereas Aslan et al. and Gormley et al. did not find any relation between the mentioned SNP and stroke morbidity among the Turkish [15] and English [16] population, respectively. Thus, the data obtained in different populations are contradictory, which requires further study about the role of polymorphic variants of the endothelin family genes in the IS development.

The aim of the present case-control study was to investigate the possible association between *EDN1* rs5370 and *EDNRA* rs5335 polymorphisms and large artery stroke (LAS) in representatives of the Ukrainian population.

## 2. Materials and Methods

### 2.1. Study Population

Venous blood of 200 unrelated Caucasians (Ukrainians) with LAS (89 females and 111 males; mean age [±SD] 66.7 ± 10.1) and 200 control subjects (75 females and 125 males; mean age 68.1 ± 13.9) was used for the study. Each stroke patient had been under dispensary observation in the 5th Sumy Clinical Hospital since April 2009 to December 2017. Computed tomography and (or) magnetic resonance imaging investigations of the head as well as electrocardiographic, biochemical, and coagulation tests and carotid ultrasonography were used for final LAS diagnosis establishment. The pathogenic variant of IS was determined according to the TOAST criteria [17]. Individuals with cardioembolic, lacunar, and hemorrhagic strokes, traumatic brain injury, and brain tumors were excluded. The clinical characteristics of LAS patients included systolic, diastolic, pulse and mean arterial blood pressure (BP), body mass index (BMI), lipid profile parameters, and coagulogram indices.

Only participants without the history of IS or other acute cerebrovascular pathologies, myocardial infarction, and other atherosclerosis complications were enrolled to the control group. Subjects of the comparison groups were divided into subgroups defined by sex and the presence or absence of AH (systolic BP > 140 mmHg, diastolic BP > 90 mmHg, or both). The study protocol complied with the Declaration of Helsinki and was approved by the Ethic Committee of the Medical Institute of Sumy State University (number 2/02.17.09). An appropriate written informed consent was obtained from all individuals.

### 2.2. Genotyping of SNPs

Genomic DNA was isolated from peripheral leukocytes using GeneJET Whole Blood Genomic DNA Purification Mini Kit (Thermo Fisher Scientific, USA). Polymerase chain reaction*-*restriction fragment length polymorphism analysis (PCR-RFLP) was used for genotyping *EDN1* rs5370 and *EDNRA* rs5335 SNPs. The reaction mixture for PCR (total volume 25 *μ*L) included 2 mM MgSO_4_, 0.2 mM dNTPs (Thermo Fisher Scientific, USA), 5 *μ*L 5 × PCR buffer, 1 U Taq DNA polymerase (Thermo Fisher Scientific, USA), and 75–100 ng DNA. The nucleotide structure of the primers and PCR conditions, which were used for each polymorphism investigation, are shown in Table 1. PСR was carried out in Thermocycler GeneAmp PCR System 2700 (Thermo Fisher Scientific, USA).

**Figure 1:**
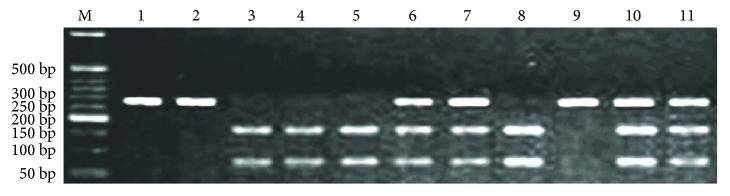
Results of *EDN1* rs5370 polymorphism restriction analysis. M—molecular marker (bp—base pairs); lanes 3, 4, 5, and 8—GG genotype; lanes 6, 7, 10, and 11—GT genotype; lanes 1, 2, and 9—TT genotype.

**Figure 2:**
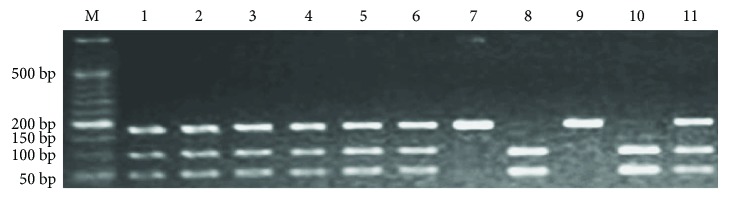
Results of *EDNRA* rs5335 polymorphism restriction analysis. M—molecular marker (bp—base pairs); lanes 8 and 10—CC genotype; lanes 1, 2, 3, 4, 5, 6, and 11—CG genotype; lanes 7 and 9—GG genotype.

2 U of *Сас*81 (Thermo Fisher Scientific, USA) was used for restriction analysis of *EDN1* rs5370 polymorphism (incubation at 37°C for 17 h). The presence of guanine at the 5665th position of the *EDN1* gene led to the cleavage of the amplicon (262 bp) by *Cac*81 into two parts of 189 and 73 bp. In the case of guanine to thymine replacement, we had only one 262 bp fragment due to the loss of the *Cac*81 catalytic site (Figure 1). Restriction analysis of *EDNRA* rs5335 SNP required using 3 U of *NmuC*I (*Tsp45*I) (Thermo Fisher Scientific, USA) (incubation at 37°C for 19 h). The presence of cytosine at the 70th position of the *EDNRA* 3′-untranslated region (3′-UTR) allowed *NmuC*I to cut the primary amplicon (174 bp) into two fragments of 116 and 58 bp. Cytosine to guanine substitution resulted in preventing the restriction and preservation of the original 174 bp fragment (Figure 2). Horizontal electrophoresis (10 V/cm) in 2.5% agarose gel (10 mg/mL ethidium bromide) with subsequent ultraviolet visualization was used for restriction fragment detection.

### 2.3. Statistical Analysis

The Statistical Package for Social Science software (SPSS, version 17.0, Chicago, IL, USA) was used for most statistical analyses. Continuous variables are presented as the mean ± SD (checking the normality of distribution was performed using Shapiro-Wilk test); categorical variables are presented as absolute number and percentage value. Two-tailed Student's *t*-test and ANOVA with subsequent Bonferroni post hoc test were used for comparison of the mean values between two or more different patient groups. In order to control type 1 error, multiple adjustment using false discovery rate (FDR) method was performed. Statistical power analysis was done using Quanto. Hardy-Weinberg equilibrium testing was carried out using Online Encyclopedia for Genetic Epidemiology Studies (http://www.oege.org/software/hardy-weinberg.html). Chi square (*χ*^2^) test was used to compare the frequency of *EDN1* rs5370 and *EDNRA* rs5335 alleles and genotypes as well as other categorical variables between the control and case groups. An odds ratio (OR) and 95% confidence interval (CI) were obtained from logistic regression for dominant, recessive, and additive models of inheritance. Multivariable logistic regression was used to exclude the effect of other atherosclerosis risk factors including sex, age, BMI, AH, type 2 diabetes mellitus (T2DM), and smoking status. All statistical tests were based on a two-tailed probability; a value of *P* < 0.05 was considered as significant.

### 2.4. Prediction Analysis

In order to uncover the functional effects of *EDN1* rs5370 and *EDNRA* rs5335 polymorphic sites, web available consensus classifiers PredictSNP2 [18] and SNPinfo were used [19]. Herewith to predict the effect of rs5370 SNP of the *EDN1* 5 exon on protein function PredictSNP was used [20]. We also used SpliceAid2 tool to check if SNP 5370 is located in splicing regulatory sequence [21]. Online miRDB resource [22] was used for microRNA target prediction in the framework of the functional analysis of rs5335 SNP of *EDNRA* 3′-UTR.

## 3. Results

The general characteristics of the study groups are summarized in Table 2. Their detailed description was presented in our previous article [23].

The distribution of *EDN1* rs5370 and *EDNRA* rs5335 alleles and genotypes in comparison groups is shown in Table 3. Obtained genotype frequencies for each SNP did not significantly deviate from Hardy-Weinberg equilibrium expectations (*P*_HWE_ > 0.05). The frequency of *EDN1* rs5370 genotypes and alleles in LAS patients significantly differed from the control group (*P* = 0.006 and *P* = 0.002, resp.), while the distribution of *EDNRA* rs5335 genotypes and alleles was similar between case and control individuals (*P* = 0.391 and *P* = 0.521, resp.). Statistical power analysis indicated that the rs5370 locus had strong power (0.689—for dominant model; 0.956—for recessive model; and 0.991—for additive model). At the same time, rs5335 SNP had poor power (0.252—for dominant model; 0.059—for recessive model; and 0.654—for additive model).

The results of two investigated SNP genotypes' association with LAS are presented in Table 4. Significant association between *EDN1* rs5370 and stroke was revealed under dominant (*P*_c_ = 0.012; OR_c_ = 1.657, 95% CI = 1.115–2.462), recessive (*P*_c_ = 0.007; OR_c_ = 2.839, 95% CI = 1.331–6.057), and additive (*P*_c_ = 0.003; OR_c_ = 3.291, 95% CI = 1.512–7.165—for TT genotype) models of inheritance. After adjusting for covariates of age, sex, BMI, AH, T2DM, and smoking status, genotypic association of rs5370 SNP remained under dominant (*P*_a_ = 0.020; OR_a_ = 1.601, 95% CI = 1.066–2.403), recessive (*P*_a_ = 0.003; OR_a_ = 3.251, 95% CI = 1.492–7.084), and additive (*P*_a_ = 0.002; OR_a_ = 3.637, 95% CI = 1.639–8.073—for TT genotype) models. Logistic regression analysis for *EDNRA* rs5335 did not show any significant link with LAS development neither before nor after adjustment for atherosclerosis risk factors (*P* > 0.05).

The analysis of rs5370 and rs5335 genotypic association with LAS risk in female and male subjects is presented in Table 5. In women, significant difference for rs5370 locus was revealed in the crude dominant (*P*_c_ = 0.021; OR_c_ = 2.090, 95% CI = 1.119–3.903) and additive (*P*_c_ = 0.034; OR_c_ = 2.008, 95% CI = 1.055–3.823—for GT genotype) models, as well as in adjusted dominant (*P*_a_ = 0.014; OR_a_ = 2.437, 95% CI = 1.202–4.940) and additive (*P*_a_ = 0.042; OR_a_ = 2.135, 95% CI = 11.079–4.393—for GT genotype; *P*_a_ = 0.018; OR_a_ = 5.634, 95% CI = 1.157–27.436—for TT genotype) models. *EDNRA* rs5335 SNP was not associated with stroke development in the female subgroup (*P* > 0.05). In male subjects, association of rs5370-TT genotype was revealed regardless of adjustment under recessive (*P*_c_ = 0.007, *P*_a_ = 0.007; OR_a_ = 3.512, 95% CI = 1.401–8.806) and additive models (*P*_c_ = 0.008, *P*_a_ = 0.009; OR_a_ = 3.535, 95% CI = 1.377–9.076). The significant link between rs5335 polymorphism and LAS development in the mentioned subgroup was absent (*P* > 0.05).

Due to influential role of the endothelin pathway in hypertension development, we also investigated the association between *EDN1* and *EDNRA* gene polymorphisms and ischemic stroke development in patients with and without AH (Table 6). In nonhypertensive subjects, both SNPs were not associated with LAS either before or after adjustment for age, sex, BMI, T2DM, and smoking (*P* > 0.05). In the hypertensive cohort, the minor T allele for the rs5370 locus was found to be significantly more prevalent in stroke patients. Before adjusting for covariates, positive association was revealed under dominant (*P*_c_ = 0.045; OR_c_ = 1.649, 95% CI = 1.011–2.689), recessive (*P*_c_ = 0.024; OR_c_ = 3.220, 95% CI = 1.164–8.903), and additive (*P*_c_ = 0.013; OR_c_ = 3.744, 95% CI = 1.322–10.607—for TT genotype) models. After adjusting for the covariates, a significant link between *EDN1* rs5370 and LAS remained under dominant (*P*_a_ = 0.036; OR_a_ = 1.711, 95% CI = 1.035–2.829), recessive (*P*_a_ = 0.008; OR_a_ = 4.102, 95% CI = 1.448–11.617), and additive (*P*_a_ = 0.004; OR_a_ = 4.743, 95% CI = 11.633–13.776—for TT genotype) models.

Table 7 indicates the clinical characteristics of LAS patients stratified by *EDN1* rs5370 genotypes. Using the ANOVA test, significant difference was found for diastolic (GG—93.4 ± 14.9 mmHg, GT—99.3 ± 16.2 mmHg, TT—91.7 ± 13.3 mmHg; *P* = 0.034) BP. Nevertheless, Bonferroni post hoc test revealed no significant difference between patients with different genotypes (*P* = 0.103 for GG versus GT; *P* = 0.087 for GT versus TT). Moreover, FDR multiple adjustment revealed that none of the parameters are associated with rs5370 genotypes. No link between *EDNRA* rs5335 genotypes and BMI, BP indices, coagulogram parameters, fasting glucose, and blood plasma lipid profile in stroke patients was found (Table 8).

The bioinformatical analysis of *EDN1* rs5370 by the SNPinfo tool showed that the mentioned polymorphic locus might be located in splicing regulatory sequences recognized by exonic splicing enhancers or exonic splicing silencers (score—2.56). SpliceAId2 tool demonstrated that rs5370 lies between recognized sites for SFRS9 and hnRNP H1 splicing factors, but this SNP has no influence on their structure. Finally, prediction analysis by PredictSNP and PredictSNP2 allowed classifying EDN1 rs5370 (Lys198Asn mutation) as neutral (PredictSNP—neutral with 83% expected accuracy (EA); MAPP—neutral with 68% EA; PhD-SNP—neutral with 78% EA; PolyPhen1—neutral with 67% EA; PolyPhen2—neutral with 61% EA; SIFT—neutral with 67% EA; SNAP—neutral with 50% EA; PredictSNP2—neutral with 89% EA; CADD—neutral with 90%; DANN—neutral with 73% EA; FATHMM—neutral with 84% EA; and FunSeq2—deleterious with 62% EA).

Prediction analysis of *EDNRA* rs5335 by the SNPinfo tool demonstrated that this SNP is possibly located in hsa-miR-27a-3p and hsa-miR-27b-3p binding sites (score—153.0). Analysis using miRDB confirmed that the *EDNRA* gene is in the list of mentioned miRNA targets (target score—50). However, PredictSNP2 results classified rs5335 mutation as neutral (PredictSNP2—neutral with 88% EA; CADD—neutral with 86%; DANN—neutral with 79% EA; FATHMM—neutral with 93% EA; FunSeq2—neutral with 62% EA; and GWAWA—deleterious with 64% EA).

## 4. Discussion

The essence of rs5370 polymorphism is the replacement of guanine by thymine at the 5665th position (5 exon) of the *EDN1* gene, which in turn leads to replacement of lysine by asparagine in the 198th position of the preproendothelin-1 molecule. Several studies have shown that endothelin-1 blood plasma concentration in T (Asn) allele carriers is higher than in subjects with GG (Lys/Lys) genotype [24, 25]. Considering the localization of this SNP, it can be assumed that its functional effect is due to the effect on the quality or speed of preprodendothelin-1 posttranslational modification. However, Tanaka et al. showed that the amount of ET-1 and big ET-1 in the supernatant of Asn-type and Lys-type transfected cells was similar [26]. In addition, the plasma endothelin-1 level in patients with essential hypertension was not different in individuals with the Asn allele and Lys/Lys genotype. The conclusion that another SNP in strong LD with rs5370 may provide its clinical effects was made. Our prediction analysis of rs5370 also did not confirm the role of this polymorphic locus in preproendothelin-1 posttranslation modification. Applying bioinformatic tools did not allow highlighting the possible functional effects of the mentioned SNP. Taking together experimental and prediction data, it seems more likely that rs5370 is in strong LD with another influential SNP.

The polymorphic site rs5335 is located within the 3′-UTR of the *EDNRA* gene and leads to cytosine/guanine conversion at position 61,772. The functional studies of this SNP do not exist, while several clinical studies have demonstrated the association of this locus with increased risk of AH development [27, 28] and level of plasma endothelin-1 [29]. On the one hand, it can be assumed that changes in the nucleotide sequence of 3′-UTR may affect the stability of mRNA and thus affect the amount of the receptor protein [30]. On the other hand, this SNP may change the structure of the miRNA binding site, as it was recently shown by Ma et al. for miR-125a and rs12976445 polymorphism of the *EDN1* 3′-UTR [31]. In order to test these hypotheses, we used bioinformatic prediction. Our results revealed that rs5335 might change the structure of hsa-miR-27a-3p and hsa-miR-27b-3p binding sites, which are the possible regulators of *EDNRA* expression. Future functional analysis to confirm these results is required.

The results obtained in the present study about the link of rs5370 (*EDN1* gene) and rs5335 (*EDNRA* gene) polymorphisms with LAS showed that in a Ukrainian population, only the rs5370 locus is associated with the development of the mentioned disease. Regardless of adjustment for other atherosclerosis risk factors, it was found that the risk of ischemic stroke development in individuals with GT and TT genotypes is higher than in GG genotype carriers. However, a similar study performed by Aslan et al. among the Turkish population did not show any correlation between *EDN1* rs5370 and rs10478694 polymorphisms and ischemic cerebrovascular disease [15]. Herewith, Gormley et al. demonstrated no association between *EDN1* (rs5370), *EDNRA* (−231G>A and +1222C>T), and *EDNRB* (G57S and 277L) gene polymorphisms and lacunar infarction development among English patients as well [16]. The authors of both studies have concluded that genetic variation in the endothelin pathway is not a risk factor for ischemic stroke.

The analysis performed in our work among subjects of different sexes has showed that rs5370 (*EDN1*), but not rs5335 (*EDNRA*) polymorphism, is associated with an increased risk of LAS both among women (under dominant and additive models) and men (under recessive and additive models). Such data were consistent with results obtained by Yamaguchi et al. [14], who assessed a possible association of 202 SNPs of 152 candidate genes with atherothrombotic cerebral infarction (ACI) in a large-scale Japanese cohort. Authors demonstrated that *EDN1* rs5370 is related to ACI development susceptibility among women (under dominant and additive models). In the men's subgroup, the link with ischemic stroke was revealed for the *EDNRA* −231A>G locus (under the recessive model). At the same time, Zhang and Sui did not reveal an association between *EDN1* rs5370 and *EDNRA* rs5335 polymorphic sites with ischemic stroke development among women of the Chinese Han population [13]. They showed that in Chinese men, the minor rs5370-T allele increased the risk of stroke morbidity, while the rs5335-CC genotype, on the contrary, was associated with a low risk of ischemic stroke incident. It should also be noted that MacClellan et al. revealed the link between *EDN1* (rs1800542 and rs10478723) and *EDNRB* (rs4885493 and rs10507875) SNPs and increased risk of ischemic stroke development in Caucasian but not in African-American women [32].

The issue of sexual difference in the endothelin system activity is one of the key topics of the cardiovascular and urinary system physiology and pathology [33]. However, the mесhаnіsm rеsроnsіblе for the dіffеrеncе іn the endothelin pathway gene SNP's association with multifactorial diseases between male and female remains unclear [14, 33]. Sex differences obtained in our study can hardly be explained by the protective effect of estrogens on the vascular wall, since the studied group included mostly postmenopausal women. It can only be said that the role of individual alleles in ischemic stroke pathogenesis undoubtedly manifested in a complex set of other important risk factors. Further studies using complex analysis to draw definitive conclusion are necessary.

To date, there are number of studies about the association between endothelin system gene polymorphisms and risk of AH and pulmonary hypertension, as well as BP indices [5, 6, 11, 24, 34–36]. The present study revealed a high risk of LAS development in rs5370-T allele carriers with AH, which is inconsistent with data obtained by Yamaguchi et al. in subgroups stratified by the presence of AH in a Japanese population [14]. Our results also demonstrated a possible correlation between rs5370 polymorphism and indices of diastolic arterial blood pressure in stroke patients. Similar results were obtained for patients with preeclampsia [24], AH [34], and healthy individuals [37]. However, the association of *EDN1* rs5370 locus with increased risk of AH development, as well as with different parameters of blood pressure among Australian patients with ischemic heart disease, was not found [11].

A significant limitation of our study is the relatively small number of patients enrolled into the case and control groups. Therefore, some associations between rs5370 and rs5335 SNPs and the risk of LAS development, indices of blood pressure, blood plasma lipoprotein level, and coagulation parameters might have been missed due to a small statistical power. Another limitation is the investigation of the link between SNPs and phenotype without an assessment of their effects on the *EDN1* and *EDNRA* mRNA level and ET-1 concentration in the blood plasma. Therefore, future case-control studies involving more patients and functional studies of endothelin pathway polymorphisms effects are required.

## 5. Conclusion

In conclusion, this is the first evidence about the association between endothelin system genetic polymorphisms and LAS development in a Ukrainian population. Our data demonstrated that *EDN1* rs5370 polymorphism is related to increased risk of LAS development regardless of other atherosclerosis risk factors. No link between *EDNRA* rs5335 and risk of LAS in a Ukrainian population was found. At present, a small number of case-control studies about the role of rs5370 and rs5335 SNPs in ischemic stroke development have been performed. Increasing the number of such studies with subsequent meta-analysis is necessary in order to draw a firm conclusion about the association between the mentioned polymorphisms and risk of ischemic stroke.

## Figures and Tables

**Table 1:** PCR conditions for *EDN1* rs5370 and *EDNRA* rs5335 genotyping.

Gene	SNP	Primer nucleotide sequence	Thermocycling conditions			PCR amplicon size
			D	H	E	
*EDN1*	rs5370	F: 5′-TCTTGCTTTATTAGGTCGGAGACC-3′ R: 5′-TTTGAACGAGGACGCTGGTC-3′	94°С (60 s)	61°С (60 s)	72°С (45 s)	262 bp

*EDNRA*	rs5335	F: 5′-TAGAAGCACTCCTCGGTACTCC-3′ R: 5′-TCG TAGATGTTGTGGGTGGATA-3′	94°С (50 s)	60°С (40 s)	72°С (60 s)	174 bp

Annotation: F: forward; R: reverse; D: denaturation; H: hybridization; E: elongation; bp: base pairs.

**Table 2:** General characteristics of the study population.

Parameter	Cases (*n* = 200)	Controls (*n* = 200)	*P*
Age, years	66.7 ± 10.1	68.1 ± 13.9	0.261
Sex, female/male	89/111	75/125	0.155
Body mass index, kg/m^2^	27.9 ± 3.7	27.3 ± 4.6	0.105
Systolic BPs, mmHg	167.9 ± 28.7	151.7 ± 22.6	<0.001
Diastolic BP, mmHg	96.0 ± 15.5	86.5 ± 11.7	<0.001
Pulse BP, mmHg	71.9 ± 22.4	65.2 ± 17.2	0.002
Mean BPs, mmHg	119.9 ± 17.9	108.3 ± 14.0	<0.001
Fasting glucose, mmol/L	6.05 ± 1.5	5.25 ± 0.7	<0.001
Total cholesterol, mmol/L	4.98 ± 1.46	4.75 ± 1.52	0.124
HDL cholesterol, mmol/L	1.01 ± 0.29	1.09 ± 0.38	0.018
LDL cholesterol, mmol/L	3.16 ± 1.39	2.94 ± 1.17	0.087
Triglyceride, mmol/L	1.67 ± 0.78	1.54 ± 0.66	0.073
Current smokers, *n* (%)	60 (30.0)	55 (27.5)	0.581
T2DM, *n* (%)	50 (25.0)	19 (9.5)	<0.001
Arterial hypertension, *n* (%)	150 (75.0)	116 (58.0)	<0.001

Categorical variables were compared by *χ*^2^ test, continuous variables by *t-*test.

**Table 3:** Distributions of genotypes and alleles in case and control groups.

Gene	SNP		LAS (*n* = 200)		Control (*n* = 200)		*P*_HWE_	*P*
			*n*	%	*n*	%		
*EDN1*	rs5370	Genotypes						
		GG	94	47.0	118	59.0	—	0.006
		GT	80	40.0	72	36.0		
		TT	26	13.0	10	5.0		
		Alleles						
		G	268	67.0	308	77.0	0.991	0.002
		T	132	33.0	92	23.0		

*EDNRA*	rs5335	Genotypes						
		CC	53	26.5	64	32.0	—	0.391
		CG	114	57.0	101	50.5		
		GG	33	16.5	35	17.5		
		Alleles						
		C	220	55.0	229	57.3	0.939	0.521
		G	180	45.0	171	42.7		

**Table 4:** Analysis of *EDN1* rs5370 and *EDNRA* rs5335 genotypic association with LAS.

Gene	SNP	Model	*P*_c_	OR_c_ (95% CI)	*P*_a_	OR_a_ (95% CI)
*EDN1*	rs5370	Dominant	0.012	1.657 (1.115–2.462)	0.020	1.601 (1.066–2.403)
		Recessive	0.007	2.839 (1.331–6.057)	0.003	3.251 (1.492–7.084)
		Additive^a^	0.096	1.426 (0.938–2.168)	0.198	1.328 (0.862–2.046)
			0.003	3.291 (1.512–7.165)	0.002	3.637 (1.639–8.073)

*EDNRA*	rs5335	Dominant	0.189	1.335 (0.867–2.056)	0.110	1.440 (0.921–2.251)
		Recessive	0.790	0.932 (0.553–1.570)	0.767	0.922 (0.539–1.578)
		Additive	0.146	1.398 (0.890–2.196)	0.078	1.522 (0.954–2.429)
			0.634	1.156 (0.636–2.103)	0.545	1.210 (0.652–2.245)

SNP: single nucleotide polymorphism; CI: confidence interval; *P*_c_: crude *P* value; OR_c_: crude odds ratio; *P*_a_: *P* value adjusted for age, sex, body mass index, arterial hypertension, type 2 diabetes mellitus, and smoking; OR_a_: adjusted odds ratio. ^a^Upper row in the additive model of inheritance—comparison between Aa and AA genotypes; lower row—between aa and AA genotypes.

**Table 5:** Analysis of *EDN1* rs5370 and *EDNRA* rs5335 genotypic association with LAS in male and female subjects.

	Model	*P*_c_	OR_c_ (95% CI)	*P*_a_	OR_a_ (95% CI)
*EDN1* rs5370					
Female	Dominant	0.021	2.090 (1.119–3.903)	0.014	2.437 (1.202–4.940)
	Recessive	0.312	2.049 (0.511–8.218)	0.086	3.824 (0.828–17.667)
	Additive	0.034	2.008 (1.055–3.823)	0.042	2.135 (1.079–4.393)
		0.149	2.852 (0.681–11.827)	0.018	5.634 (1.157–27.436)
Male	Dominant	0.231	1.371 (0.818–2.297)	0.236	1.375 (0.812–2.330)
	Recessive	0.007	3.481 (1.404–8.635)	0.007	3.512 (1.401–8.806)
	Additive	0.939	1.022 (0.581–1.800)	0.952	1.018 (0.571–1.815)
		0.008	3.510 (1.382–8.913)	0.009	3.535 (1.377–9.076)

*EDNRA* rs5335					
Female	Dominant	0.220	1.523 (0.778–2.981)	0.065	2.012 (0.956–4.230)
	Recessive	0.273	1.579 (0.698–3.572)	0.351	1.515 (0.633–3.629)
	Additive	0.352	1.398 (0.691–2.831)	0.105	1.921 (0.873–4.226)
		0.159	1.953 (0.770–4.952)	0.111	2.256 (0.829–6.136)
Male	Dominant	0.482	1.224 (0.696–2.153)	0.514	1.210 (0.682–2.148)
	Recessive	0.172	0.607 (0.297–1.242)	0.217	0.631 (0.304–1.310)
	Additive	0.257	1.405 (0.780–2.529)	0.299	1.373 (0.755–2.494)
		0.505	0.758 (0.336–1.710)	0.549	0.776 (0.339–1.777)

See Table 4; *P*_a_: *P* value adjusted for age, body mass index, arterial hypertension, type 2 diabetes mellitus, and smoking.

**Table 6:** Analysis of *EDN1* rs5370 and *EDNRA* rs5335 genotypic association with LAS in subjects with and without arterial hypertension.

	Model	*P*_c_	OR_c_ (95% CI)	*P*_a_	OR_a_ (95% CI)
*EDN1* rs5370					
Nonhypertensive	Dominant	0.262	1.500 (0.739–3.046)	0.201	1.624 (0.773–3.411)
	Recessive	0.125	2.572 (0.770–8.593)	0.118	2.685 (0.778–9.269)
	Additive	0.556	1.259 (0.584–2.714)	0.470	1.348 (0.600–3.029)
		0.104	2.800 (0.810–9.680)	0.093	2.980 (0.834–10.650)
Hypertensive	Dominant	0.045	1.649 (1.011–2.689)	0.036	1.711 (1.035–2.829)
	Recessive	0.024	3.220 (1.164–8.903)	0.008	4.102 (1.448–11.617)
	Additive	0.188	1.411 (0.845–2.354)	0.212	1.400 (0.825–2.374)
		0.013	3.744 (1.322–10.607)	0.004	4.743 (1.633–13.776)

*EDNRA* rs5335					
Nonhypertensive	Dominant	0.170	1.793 (0.779–4.125)	0.132	1.962 (0.817–4.717)
	Recessive	0.443	1.457 (0.558–3.806)	0.531	1.371 (0.512–3.672)
	Additive	0.218	1.715 (0.727–4.048)	0.159	1.909 (0.776–4.698)
		0.196	2.127 (0.678–6.676)	0.198	2.190 (0.664–7.221)
Hypertensive	Dominant	0.386	1.260 (0.747–2.126)	0.271	1.353 (0.790–2.318)
	Recessive	0.325	0.730 (0.390–1.366)	0.317	0.718 (0.376–1.373)
	Additive	0.214	1.420 (0.816–2.471)	0.135	1.545 (0.873–2.735)
		0.788	0.970 (0.445–1.850)	0.864	0.938 (0.449–1.958)

See Table 4; *P*_a_: *P* value adjusted for age, sex, body mass index, type 2 diabetes mellitus, and smoking.

**Table 7:** Characteristics of the LAS subjects stratified by *EDN1* rs5370 genotype.

Parameter	Genotype			*P* value	*P*_FDR_
	GG (*n* = 94)	GT (*n* = 80)	TT (*n* = 26)		
Body mass index, kg/m^2^	28.1 ± 3.6	27.6 ± 3.8	28.4 ± 3.9	0.448	0.647
Systolic BP, mmHg	165.1 ± 29.3	171.3 ± 28.8	167.5 ± 25.9	0.372	0.642
Diastolic BP, mmHg	94.4 ± 14.9	99.3 ± 16.2	91.7 ± 13.3	0.034^a^	0.429
Pulse BP, mmHg	70.7 ± 20.2	71.9 ± 24.8	75.8 ± 22.5	0.600	0.720
Mean BP, mmHg	117.9 ± 18.5	123.3 ± 17.8	116.9 ± 15.2	0.099	0.429
Total cholesterol, mmol/L	4.91 ± 1.4	5.15 ± 1.6	4.74 ± 1.3	0.374	0.641
HDL cholesterol, mmol/L	1.02 ± 0.31	1.01 ± 0.31	0.96 ± 0.25	0.610	0.720
LDL cholesterol, mmol/L	3.10 ± 1.3	3.33 ± 1.5	2.89 ± 1.3	0.347	0.642
Triglyceride, mmol/L	1.65 ± 0.8	1.71 ± 0.8	1.58 ± 0.7	0.750	0.812
Prothrombin time, s	9.47 ± 2.0	9.38 ± 2.0	9.29 ± 2.0	0.900	0.900
Thrombin time, s	16.71 ± 3.7	16.49 ± 3.5	17.60 ± 4.0	0.395	0.642
Fibrinogen, g/L	4.21 ± 1.3	3.93 ± 1.2	3.64 ± 1.3	0.089	0.429
Fasting glucose, mmol/L	6.17 ± 1.7	6.06 ± 1.4	5.59 ± 1.0	0.236	0.642

*n*: number of cases; BP: blood pressure; HDL: high-density lipoprotein; LDL: low-density lipoprotein; *P*_FDR_: *P* value after false discovery rate adjustment. ^a^Nonsignificant difference between GG and GT genotypes (*P* = 0.103) and between TT and GT genotypes (*P* = 0.087) by Bonferroni post hoc test.

**Table 8:** Characteristics of the LAS patients stratified by *EDNRA* rs5335 genotype.

Parameter	Genotype			*P* value	*P*_FDR_
	CC (*n* = 41)	CG (*n* = 98)	GG (*n* = 31)		
Body mass index, kg/m^2^	28.7 ± 3.3	27.7 ± 3.8	27.6 ± 3.6	0.251	0.566
Systolic BP, mmHg	169.5 ± 26.9	167.3 ± 28.6	167.3 ± 32.2	0.888	0.914
Diastolic BP, mmHg	96.9 ± 12.7	96.7 ± 16.0	91.9 ± 17.3	0.261	0.566
Pulse BP, mmHg	72.6 ± 21.4	70.6 ± 21.6	75.3 ± 26.5	0.548	0.745
Mean BP, mmHg	121.2 ± 15.6	120.2 ± 18.5	117.1 ± 19.7	0.573	0.745
Total cholesterol, mmol/L	5.18 ± 1.6	5.01 ± 1.4	4.64 ± 1.4	0.257	0.566
HDL cholesterol, mmol/L	0.95 ± 0.31	1.02 ± 0.30	1.07 ± 0.27	0.179	0.566
LDL cholesterol, mmol/L	3.37 ± 1.5	3.18 ± 1.4	2.83 ± 1.3	0.235	0.566
Triglyceride, mmol/L	1.78 ± 0.8	1.66 ± 0.8	1.54 ± 0.7	0.383	0.671
Prothrombin time, s	9.81 ± 2.2	9.26 ± 1.9	9.31 ± 1.9	0.252	0.566
Thrombin time, s	16.63 ± 3.2	16.72 ± 3.6	16.96 ± 4.3	0.914	0.914
Fibrinogen, g/L	3.95 ± 1.5	4.01 ± 1.2	4.00 ± 1.3	0.878	0.912
Fasting glucose, mmol/L	6.29 ± 1.8	5.99 ± 1.3	5.89 ± 1.5	0.413	0.671

See Table 7.
